# Eliciting and Representing High-Level Knowledge Requirements to Discover Ecological Knowledge in Flower-Visiting Data

**DOI:** 10.1371/journal.pone.0166559

**Published:** 2016-11-16

**Authors:** Willem Coetzer, Deshendran Moodley, Aurona Gerber

**Affiliations:** 1 SAIAB: South African Institute for Aquatic Biodiversity, Private Bag 1015, Grahamstown 6140, South Africa; 2 CAIR: Centre for Artificial Intelligence Research, CSIR Meraka, PO Box 395, Pretoria, 0001, South Africa; 3 Department of Informatics, University of Pretoria, Private Bag X20, Hatfield, 0028, South Africa; 4 Department of Computer Science, University of Cape Town, Private Bag X3, Rondebosch, 7701, South Africa; 5 School of Mathematics, Statistics and Computer Science, University of KwaZulu–Natal, Private Bag X54001, Durban 4000, South Africa; Oregon State University, UNITED STATES

## Abstract

Observations of individual organisms (data) can be combined with expert ecological knowledge of species, especially causal knowledge, to model and extract from flower–visiting data useful information about behavioral interactions between insect and plant organisms, such as nectar foraging and pollen transfer. We describe and evaluate a method to elicit and represent such expert causal knowledge of behavioral ecology, and discuss the potential for wider application of this method to the design of knowledge-based systems for knowledge discovery in biodiversity and ecosystem informatics.

## Introduction

Biodiversity scientists and ecologists work in different sub–domains including taxonomy, community ecology, behavioral ecology, conservation planning and many others. The analytical methods and knowledge production processes [1,2] used in these different sub–domains are common to all natural sciences. The scientific method will be employed to eliminate or minimize variability and uncertainty in order to test a hypothesis. Frequentist [3] or Bayesian [4] statistical analysis of empirical observations will then be conducted and the process will culminate in publication of conclusions in the primary literature. In the field of flower–visiting ecology the process of knowledge production typically starts with analyses of observations of interacting plants and animals, either drawn from legacy natural history collection data [5] or collected *de novo* during field surveys [6]. At this point an expert can generate knowledge according to the traditions of natural science, by manually summarizing and analysing these data and interpreting the results using available or personal knowledge. In the work described below we report on a method that we developed to elicit and represent higher-level knowledge typically called upon by ecologists to reason with, and interpret, their data. Our objective is to advance techniques for discovering ecological knowledge in databases through knowledge engineering [7].

Whereas several ecology and biodiversity [7,8] ontologies have been created in the field of biodiversity and ecosystem informatics (BDEI), techniques and applications that use ontologies in ecological knowledge engineering are still developing. An ontology was used to synthesize new conceptual ecological models from metadata in datasets by matching an existing model with input metadata concepts constrained by the ontology [9]. Several ontologies have been created for ecoinformatics, namely an ecology ontology as well as ontologies for ecological models, analysis methods, ecological networks, and observations and measurements. These can be used to describe ecological and environmental data to facilitate their discovery in particular contexts, and to describe data analysis tools to create scientific workflows [1,7,10–13].

In previous work [14], upon which we build in the work reported below, we developed an ontology framework as part of a system that performs semantic enrichment and improves semantic interoperability between heterogeneous records of flower–visiting observations. The context is natural history specimen–records (e.g. of bees) in museum data–stores, which are legacy data digitised from specimens with small labels which can be packed efficiently into storage drawers. These digitised labels represent incomplete information, including about the ecological association between each flower–visiting specimen (e.g. insect) and the plant on which the insect had been captured in the field. An expert flower–visiting ecologist can discern which specimen–records represent situations where pollination is likely to have taken place or at least where the requirements for potential pollen transfer were met. Our objective was to combine the incomplete label information in each specimen–record with domain knowledge in a way that simulates the inferencing ability of a group of experts—to detect behavioral interactions and potential pollen transfer.

Previously [14] we had defined a class at a high level of abstraction, namely *ArthropodPlantInteraction*. This class represented instances when an individual insect and plant were deemed to have been involved in an interaction, which we now term a behavioral interaction (class *BehavioralInteraction*). We previously defined various kinds of low-level events subsumed by the class *PlantAssociationEvent*, an instance of which is a *part_of* an instance of the class *ArthropodPlantInteraction*. These events mainly represent the movements or behavior of arthropods on or near flowers, recorded by scientists in detailed observations. For example, when pollen is transferred, either from the anther to the arthropod vector or from the vector to the flower’s stigma, there is an instance of the class *FlowerPollenTransferEvent*. The process of pollen transfer itself is, however, not frequently or readily observed, except perhaps in certain families of plants that produce large pollinaria which adhere to certain large insects. Similarly insect foraging behavior is difficult to observe. Unless exclusion trials are conducted [15], ecologists studying flower–visiting therefore usually need to infer that foraging or pollen transfer took place. It is this inferencing that we seek to automate.

Detailed observations of the behavior of flower–visiting arthropods on flowers, alone, can be used to infer that foraging or pollen transfer took place. If these inferences are to be reliable, however, other relevant fields in the specimen–record as well as relevant available knowledge need to be combined in a way that simulates the way in which a domain expert would implicitly model knowledge and reason with the combined data and knowledge. For example, our semantic enrichment system [14] allows a record of an association between an arthropod and a plant to be extracted and enriched as a special flower–visiting behavioral interaction as long as the arthropod belongs to a known flower–visiting taxonomic group such as bees (since natural history specimen–records often omit important behavioral detail such as whether the arthropod was actually observed on the flower whose species name appears on the specimen label). Clearly this would be incorrect, however, if the available knowledge is that the plant species in question is typically not in flower (represented by the class *FloweringTime*) at the time of the year when the observation was made. This prompted us to ask: What other knowledge, relating to the factors affecting foraging and pollen transfer, in particular, need to be considered, and how should they be combined in a model? We conducted an exploratory exercise in eliciting and modeling ecological knowledge held by expert ecologists and reflected a conceptual model of their knowledge back to them for evaluation.

### Modeling choices

Whereas the semantic mediation system [14] was useful for semantic enrichment and dynamic integration of heterogeneous data, it could not tell us what insects were probably doing on flowers. The events we previously modeled, e.g. instances of the class *FlowerNectarIngestingEvent*, were relatively low–level representations. To recognise and understand an unfolding ecological process (e.g. the process generally termed a plant–insect interaction in the domain) we needed a composite class combining such a low–level event with expert knowledge and other contextual data. In other words we needed to represent a situation. For this reason we followed the general approach used in situation awareness, which encapsulates knowledge of the relative positioning of, or relationships between, objects, or how the current state of the world is comprehended [16]. Such high–level information is more useful to an ecologist who sees or projects the world not as a collection of static objects requiring classification (which might suffice in taxonomy) but as dynamic processes e.g. the flow of information (DNA) or energy, or flow of a substance such as a nutrient or pollutant [1,17], or an interaction between species. We therefore re–approached our case study [14] from a different point of view, namely that of the expert who understands the factors affecting the causal relations between ecological events, and modelled the knowledge using a semantic Bayesian network (BN) [18].

A Bayesian network (Bayes net or Bayesian belief network) is a model that graphically and probabilistically represents correlative and causal relationships among variables [18,19]. A BN has two types of nodes: observation or measurement nodes and inferred nodes, connected by arcs representing causal influences. A BN node is implicitly understood to be an event which can be in one of a number of states at a given time. To specify the probability distribution of a BN, one ‘must give the prior probabilities of all root nodes (nodes with no predecessors) and the conditional probabilities of all nonroot nodes given all possible combinations of their direct predecessors’ [18].

The BN formalism reflects the event–centric perspective on ecology developed in our previous work. Furthermore, the dependency–chain of consequent events inherent in a BN model can easily be translated into an ecological network, a modeling artefact that is currently popular in flower–visiting studies.

### Scope of modeling

There are many reasons why arthropods are attracted to or land on flowers, or repeatedly visit flowers by flying from flower to flower. We modelled the three behavioral interactions that distinguish the more specialised anthophilous (flower–visiting) insect species (usually bees, or the superfamily Apoidea, and masarine wasps, or the subfamily Masarinae) from other arthropods that can be found on flowers but are not typical flower–visitors. These behaviors are active foraging for nectar, active foraging for pollen (with or without vibrating the wings to release poricidally dehiscent pollen), active foraging for oil and the passive transfer of pollen that is an incidental consequence of these behaviors. In our conceptual model the only other relevant event is a generalized *FlowerUtilizingEvent* which takes place when an arthropod utilizes a flower for any reason (e.g. chewing and ingesting the flower parts, concealment, protection, finding a mate or laying eggs), including that of foraging for a floral product. In other words we did not model behaviors other than those associated specifically with foraging for floral products.

We focused on interpreting data relevant to inferred behavioral interactions between individual organisms (i.e. between an insect of *species A* and a plant of *species B*). Evidence used for inferencing originated on the insect specimens’ labels, where a note contains the name of a plant species (at least) (i.e. a *PlantAssociationEvent*) and sometimes more–detailed information such as how the insect was behaving in relation to the plant’s flowers (e.g. ‘feeding on nectar’) before the insect was captured and preserved.

We limited our knowledge modeling to preserved museum specimens of arthropods collected in Africa, thereby excluding behavioral interactions exhibited by anthophilous arthropods found outside Africa (e.g. arthropods that collect fragrances from flowers to attract mates). We excluded observations that are not linked to preserved museum specimens because we plan, in future work, to enumerate and aggregate records of the same species into population samples, and must therefore be certain that different database records represent different individual organisms (each labelled with unique museum catalogue numbers).

### Objectives

Our ultimate objective is to design a knowledge–based system for high–level reasoning to simulate the combined inferencing ability of a group of domain experts. The system would automate the identification of situations of interest among flower–visiting records, specifically to infer or detect behavioral interactions (e.g. foraging for nectar or pollen transfer). From our interactions with experts we deemed the combination of discrete knowledge (modelled in an ontology) and probabilistic, causal knowledge (modelled in a BN) potentially to be more useful in our application than an ontology alone. Our contribution consists of the method we developed to elicit and represent expert causal knowledge, the conceptual model itself, and the reflection upon our experience and what we learned from the exercise. The following description and discussion therefore detail the BN modeling work towards our ultimate objective. Further ontology development and implementation in a prototype system is left for future work.

Broadly, we elicited experts’ natural language sentences containing causal knowledge of the factors affecting the inferences experts draw from their flower-visiting observations (data). We then abstracted the necessary knowledge elements from these elicited natural language sentences to represent and formalize these as knowledge requirements. We combined random variables representing the knowledge elements in a semantic BN. The final step was to evaluate the semantic BN through qualitative feedback from experts.

Knowledge elicitation and modeling steps:

1. Elicit natural language statements from experts, describing the behavioral and ecological factors that affect an expert’s belief that a behavioral interaction (e.g. foraging for nectar) occurred, given the available data;
2. Identify the knowledge elements, or select, among these natural language statements, the kinds of observations and knowledge that are important, and classify and characterize these;
3. Formalize or represent the knowledge elements as high–level Knowledge Representation and Reasoning (KRR) requirements, and develop the random variables and BN;
4. Refine and evaluate, through expert feedback, the BN as a model to represent expert causal knowledge.

## Method and Results

The present work is an exception among research undertaken by staff of the South African Institute for Aquatic Biodiversity (SAIAB) and was deemed not to require the approval of the SAIAB Ethics Committee. Experts whose knowledge was elicited consented, in writing, to participate in this study.

### Eliciting expert knowledge in natural language

We consulted (S1 File) five experts in flower–visiting ecology and asked them what kinds of behavioral interactions involving flower–visitors and flowers are recognized. We also asked them, if given a flower–visiting record, what factors affect their belief that a specific flower–visiting behavioral interaction, including pollen transfer, took place.

Using this information a BN was created and given to the experts as a way to focus their attention on the factors that allow them to assert, when looking at their data, that these flower–visiting behavioral interactions and pollen transfer took place. This elicitation process resulted in new expert knowledge to incorporate into the BN model because experts understood how the model simulated their thinking.

An expert with more than 30 years’ experience of the foraging and pollinating behavior of flower–visiting insects was further consulted to elicit more–detailed knowledge. This expert’s knowledge was captured as natural language assertions e.g.

It is likely that pollen transfer occurred

 if the arthropod–plant relationship is an obligate mutualism

 and if the observation of the arthropod–plant relationship was made during the flowering period

 or

 if the arthropod is a female bee or female pollen wasp

 and if the observation of the arthropod–plant relationship was made during the flowering period

### Identifying and characterizing the knowledge elements

The knowledge elements contained in the natural language sentences were identified and rewritten as random variables (summarized in Table 1). The random variables were classified as observations (i.e. data) or knowledge or inferences, and categorized into kinds of knowledge more–or–less corresponding to fields in biodiversity science or ecology.

**Table 1:** The random variables extracted from natural language sentences elicited from experts.

Knowledge element	Kind of knowledge	Random variable
Observation	Molecular / Microscopic	Pollen evidence (free pollen)
Observation	Curatorial i.e. a plant name appears on an insect label	FlowerAssociation
Observation	Behavioral / Ecological	Duration of visit
Observation	Behavioral / Ecological	Observed behavior: Utilizing a flower
Observation	Behavioral / Ecological	Observed behavior: Foraging for a floral product
Observation	Behavioral / Ecological	Observed behavior: Vibratory pollen collection
Observation	Behavioral / Ecological	Observed behavior: Foraging for pollen
Observation	Behavioral / Ecological	Observed behavior: Foraging for nectar
Observation	Behavioral / Ecological	Observed behavior: Foraging for oil
Observation	Behavioral / Ecological	Robbing nectar (piercing the corolla to get nectar)
Observation	Behavioral / Ecological	Thieving nectar (removing nectar without piercing)
Observation	Anatomical / Morphological	Sex
Inference or observation	Behavioral / Ecological	Pollen transfer (vector–receiving)
Inference	Behavioral / Ecological	Visit to different flower of same species
Inference	Behavioral / Ecological	Pollen transfer (stigma–receiving)
Knowledge	Molecular / Microscopic	Pollen identification reference
Knowledge	Anatomical / Morphological	Known oil–producing plant species
Knowledge	Anatomical / Morphological	Plant species producing pollen only
Knowledge	Anatomical / Morphological	Poricidal dehiscence
Knowledge	Anatomical / Morphological	Plant species has Insect Pollination Syndrome
Knowledge	Anatomical / Morphological	Flower size
Knowledge	Anatomical / Morphological	Inflorescence type: Simple or flat compound vs. compound
Knowledge	Ecological	Plant species known to be robbed
Knowledge	Ecological	Plant species known to be thieved
Knowledge	Ecological	Collecting date is within flowering period
Knowledge	Ecological / Morphological	Known oil collecting vector species
Knowledge	Morphological	Vector size
Knowledge	Ecological / Behavioral	Known vibratory pollen foraging vector species
Knowledge	Ecological / Behavioral	Vector behavior
Knowledge	Ecological	Known thieving arthropod species
Knowledge	Ecological	Known robbing arthropod species
Knowledge	Ecological	Known pollen–specialist vector species
Knowledge	Ecological	Degree of oligophagy
Knowledge	Ecological	Independent evidence of flower–visiting
Knowledge	Anatomical / Morphological	Known nectar-producing plant species

We related the kinds of knowledge represented in the semantic BN to fields of biodiversity science and ecology and noted the sources of knowledge in these fields (Table 2).

**Table 2:** The fields of biodiversity science or ecology which give rise to the concepts represented by the BN random variables.

Kind of knowledge	Source of knowledge	Field of biodiversity science or ecology
Knowledge of molecular biology	Online databases containing reference gene sequences	Gene sequencing or DNA barcoding
Curatorial and natural history knowledge (biological/ecological annotations on specimen labels)	Specialized natural history collection databases	Natural history collection management and curation, or biodiversity informatics
Behavioral / ecological knowledge	Specialized techniques, field surveys, projects, publications e.g. [20],[21] and experts	Behavioral ecology or community ecology
Morphological knowledge (including the microscopic level)	Specialized techniques, projects, publications (e.g. containing pollen micrographs) and experts	Microscopic analysis of pollen
Anatomical / morphological knowledge	Specialized publications e.g. [20], online repositories (including DL knowledgebases) and experts	Systematics and taxonomy

Further, we highlighted the kinds of observations and knowledge that are most useful in causal knowledge representation and reasoning in the analysis of flower–visiting biodiversity occurrence records. These are behavioral and ecological as well as taxonomic knowledge elements, for example:

- whether an insect species belongs to a known flower–visiting group such as bees (taxonomic knowledge);
- the specific type of flower–visiting relationship, i.e. whether an arthropod is a nectar and pollen feeder, a specialist oil collector or a specialist pollen collector (behavioral ecology);
- the type of floral reward, i.e. only pollen, pollen and nectar, or pollen and oil (ecological knowledge);
- whether a plant species is known to flower during a particular month (ecological knowledge), i.e. when it is not known that an insect was observed on a flower, but some association between an insect specimen and a plant is implied by the appearance of the plant species name on the insect specimen label (which is a unique combination of knowledge and data found in natural history collections and the experts associated with collections).
- The degree of oligophagy, or how many species of plants an insect visits to obtain nectar, which affects the chance that a given insect will visit another plant of the same species for nectar, and thereby transfer pollen (behavioral ecology).

### Formalizing the high–level KRR requirements and creating a consensus BN

The natural language sentences were formalized into standard, semi–formal assertions e.g.

It is: [degree of probability]

  that [*behavioral interaction*] occurred (**event 1**)

   if [*combination of causal biological factors* exists i.e. observations and knowledge]

    and consequently it is

 [degree of probability]

  that a pollen transfer *behavioral interaction* occurred (**event 2**)

We then specified the high–level KRR requirements in the analysis of flower–visiting behavioral ecology data:

1. the variables included in the BN model;
2. the class *BehavioralInteraction*, an instance of which is a behavioral interaction between two organisms (an event). This class has the sub–classes *ForagingForNectar*, *ForagingForPollen*, *ForagingForOil* and *PassivelyTransferringPollen*; A formal definition of the class *BehavioralInteraction* will be developed in future work;
3. a situation, which is a state of a given BN at a point in time, considering all available knowledge, observations and beliefs e.g. the probability that a *ForagingForNectarSituation* took place;

### Refining and evaluating the Bayesian network

**Fig 1:**
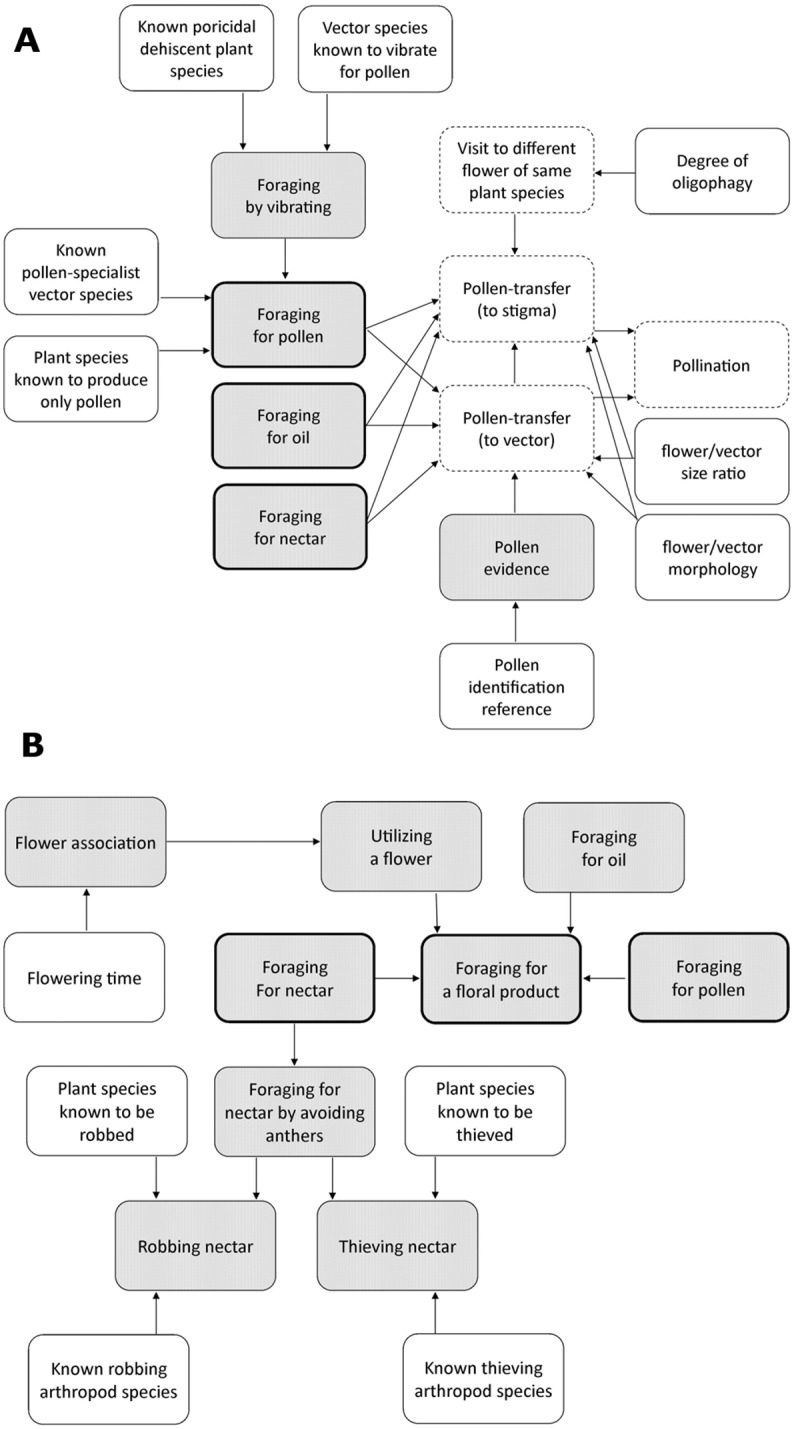
The refined BN, divided into two parts for easier display—the nodes representing *Foraging for nectar*, *Foraging for oil* and *Foraging for pollen* (heavier borders) appear in both parts to allow them to be integrated. Shaded nodes represent data and unshaded nodes represent knowledge. Nodes with dashed borders are nodes that can only be inferred and nodes with solid borders are evidence nodes, which can also be inferred.

We created a BN to represent a reasonable consensus of experts. The data from twelve typical flower–visiting records were then used to set the evidence nodes in the BN and evaluate the posterior probability of behavioral interactions and pollen transfer for each record. The results were compiled and presented to the five flower–visiting experts, who were asked to evaluate the results and comment on whether the BN was a reasonable model. All five experts concurred that the results were reasonable, but all five experts also made comments which resulted in refinement of the model. We further consulted a flower–visiting and pollination expert, who added new, significantly more-detailed knowledge to the BN. The refined BN is shown in Fig 1a and 1b. When implemented, the BN will receive a specimen-record (i.e. a digitised specimen label) as input from a data-store. Such a record would already represent an instance of the class *PlantAssociationEvent* in the flower–visiting ontology [14] because there would be a plant name on the specimen label. The BN would then evaluate the posterior probabilities of events represented by the nodes *ForagingForNectar*, *ForagingForPollen*, *ForagingForOil* and *PassivelyTransferringPollen*.

The table of prior probabilities associated with the node representing one of the BN variables is shown in Table 3. The degree of oligophagy of an arthropod species was deemed to be the most important variable affecting the belief that a given insect would visit a second flower of the same plant species, a prerequisite of pollen transfer. This is a good example of the kind of causal knowledge of behavioral ecology that needs to be represented to extract useful information from ecological data.

**Table 3:** The conditional probability table associated with the BN node representing the variable *Visit to Second Flower of the Same Species*.

*Degree of oligophagy*	Conditional probability of *Visit to Second Flower of the Same Species*
Obligate mutualist or female Colletidae or female Melittidae or female Masarinae	55%
Female bee other than Colletidae or Melittidae, or male bee or male masarine wasp or independent evidence of flower–visiting to limited number of species	30%
Nectar feeding flower–visitor other than the above	15%
Flower–visitor that is not a nectar feeder	0

### Reflection on the Validity and Usefulness of the Method and BN

Reflecting on the semantic BN as a tool for knowledge elicitation and representation, we found that representing causal ecological knowledge enabled us to model behavioral interactions and estimate the probability associated with their occurrence. The formalism we chose was also useful as an elicitation method because experts were intuitively able to interrogate and tease apart composite, high-level events and situations, using causality as a mechanism. Indeed, whereas reactions to the ontology framework and semantic mediation system [14] were somewhat mixed, ecologists could more easily relate to the objective of replicating, using a computer, the way that they reason with their own knowledge and data. This could be an important area for future research because potentially it represents the key to unlocking biodiversity and ecology KRR. In other words, modeling expert knowledge using a semantic BN could be a way to reduce the complexity of expert knowledge without the need for discrete representational classes, at least as a first step in knowledge modeling.

### Conceptual stance

One of our findings was that a conceptual stance or perspective on ecology and ecological interactions was needed in order to usefully and consistently represent the implicit expert knowledge used in inferencing. The methodological status of ecological concepts is still characterized by ambiguity and terminological confusion i.e. ‘many synonyms exist for the same ecological unit and there are cases where the same term is used for different concepts’ [22] e.g. the terms for the units ‘population’, ‘community’, and ‘ecosystem’, and the term ‘ecological interaction’. Many terms have not enjoyed formal scrutiny. For example, the ecological or species interaction colloquially termed ‘pollination’ has been classified as a ‘non–trophic species interaction that modifies non–feeding parameters, specifically reproduction’ [23], a definition that calls into question the meaning of several other concepts.

**Fig 2:**
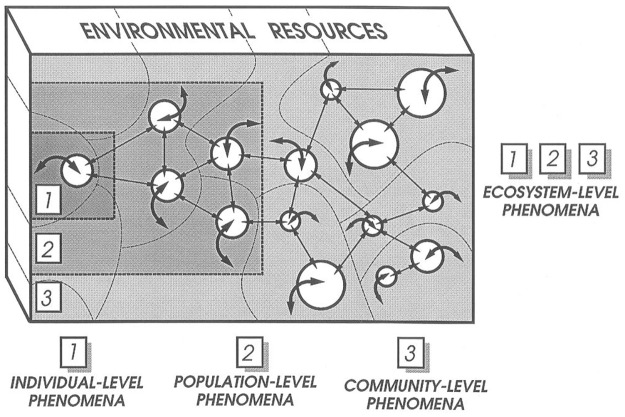
Graphical conceptualization of an individual–based computational model illustrating how individual–level processes produce patterns at higher levels of complexity. Different sizes of circles (organisms) represent different species. Broad arrows represent feedback between organisms and the environment (also a mechanism of indirect interaction between organisms) and thin arrows represent direct interactions between individual organisms. (Huston, M., DeAngelis, D. and Post, W. 1988. New computer models unify ecological theory. Bioscience 38(10): 682–691 by permission of Oxford University Press).

Whereas the concept of an *ecological interaction* was an implicit knowledge requirement (of fundamental importance in BDEI) that remained unstated by the experts we consulted, they articulated other high–level knowledge, specific to flower–visiting ecology, in detailed terms e.g. the behavior of a single bee. If an individual bee behaves in a specific way on a flower there is said to be an instance of the class *BehavioralInteraction* between the bee organism and the plant organism, though the word ‘interaction’ is not meant to indicate that the instance has any properties in common with an instance of the putative class *EcologicalInteraction*. The word ‘interaction’ in the class *BehavioralInteraction*, therefore, merely means that the individual bee and plant are moving or behaving or acting (interacting) ‘with or towards each other’ in concrete terms and can be observed to be doing so. Our concept of a behavioral interaction between individuals is broadly consistent with the conventional perspective on ecology [24], which recognizes the individual, population and community levels as the salient levels of ecological organisation, and the individual as the ‘currency unit’. Working at the intersection of ecology and computational modeling of complex systems Huston et al. [25] discussed the application of individual–based computational models to studies of populations, communities and ecosystems as well as feeding and predation (ecological interactions). The three levels of ecological organization were depicted to illustrate how individual–level processes produce patterns at higher levels of organisation [25] (Fig 2). The argument is that the individual organism is the logical basic unit for modeling ecological phenomena, as the use of aggregated state–variables in population models, for example, makes the simplifying assumption that all individuals are statistically similar and interact similarly with other organisms and the environment. Small individual differences, however, can lead to significant effects at higher levels of organization [25].

### The domain perspective on flower–visiting

Data on whether or not a particular insect visited a second flower of the same species, or whether the direction of pollen transfer was ‘vector-receiving’ or ‘stigma–receiving’, are not available in typical natural history specimen-records. Some authors claim that flower–visiting data are a poor proxy for pollination [26], noting that some of the most important factors affecting pollination are the duration of the visit, the frequency of visits to a given flower (from a flower’s perspective)[6] and the behavior of the vector in the flower. Typical natural history specimen-records, however, document the insect’s perspective as a once–off observation (after which the insect is killed and preserved), precluding the collection of such quantitative data or data from a single, monitored flower which is visited many times. Even the behavior of an insect on a flower represents detail that is included in only the most specialized natural history research projects and databases. Experts nevertheless concurred within the scope of our application, which is limited to the particular context of discovering knowledge from typical (if unusually rich) natural history specimen-records such as they are.

#### The quality of knowledge elements

The quality of knowledge elements, including the veracity of generally available (often implicit) knowledge and the provenance of data, have a bearing on evaluating the BN as a modeling tool. We elicited and modeled highly detailed, if not comprehensive, knowledge of flower–visiting and pollination, and combined this knowledge with unusually rich natural history specimen–records. The specimen-records are the legacy of Dr F.W. Gess and Dr S.K. Gess of the Albany Museum and are noteworthy for their detail, specifically with respect to insect behavior and the flowers visited by bees and wasps [21].

Reflecting on the techniques employed in biodiversity knowledge discovery in databases can inform fieldworkers as to what kinds of data are needed to more easily enrich a database record with, or extract from it, as much meaning and value as is possible. In many cases the semantic enrichment need not be as detailed as the described case of flower-visiting behavioral interactions and pollen transfer. Even making the simple assertion that two organisms were involved in a generalized behavioral interaction could significantly and meaningfully increase the amount of information available for traditional biodiversity data analyses or ecological knowledge engineering.

#### Reasoning about the degree of oligophagy and reasoning about oil–collecting by specialist bees

There is a need to distinguish between pollen collected for nest provisioning (i.e. pollen as food for larvae developing in the nest) and free pollen [20]. Whereas the former is actively ingested into the crop or packed into external pollen–carrying structures for transport, the latter accidentally adheres to the insect when it is searching for nectar, which is food for the adult insect. The pollen that is transferred between flowers and ultimately fertilizes the ovum is free pollen. An oligolectic bee species is one that collects and transports to its nest, as provision, the pollen of only a few plant species (say, fewer than 10 species). An oligophagous bee species, on the other hand, is one that feeds on the nectar of only a few plant species i.e. for its own energy needs. A flower produces just enough nectar to attract a bee but not enough to satisfy it, thereby forcing the bee to find another flower [27]. It is this tendency of females (males are not as long lived) of certain bee families, and female pollen wasps (family Masarinae), to go from flower to flower of the same plant species, or a limited number of species, in search of nectar, that most predictably causes free pollen to be transferred from one flower’s anther to another conspecific flower’s stigma. For this reason the degree of oligophagy (not oligolecty) exhibited by an insect species strongly influences an expert’s belief that it may transfer pollen between conspecific flowers. Similar reasoning applies to the case of oil–collecting bees, which collect oil from particular oil-producing plant species: if plants of a small number of species are visited the chance of pollination is higher than if plants of many species are visited [28].The degree of oligophagy is perhaps the most important knowledge element in the BN. Knowledge of the degree of oligophagy of insect species has been collected, compiled and published for more than a century, and is included in specialized texts such as reference books [20] and journal articles (including reviews on the subject, such as 27), and is therefore generally available. This knowledge was both easy to elicit and easy to represent due to its discrete nature (Table 3) and the availability of experts.

#### Reasoning about pollen evidence

Similarly the presence or absence of pollen evidence, or pollen found on the insect’s body and identified through microscopy [29] or DNA barcoding is also an important factor influencing the belief that pollen was transferred, at least from the anther to the insect vector. If a field worker used a single collecting net or killing bottle to contain more than one insect specimen there is a possibility (nevertheless implicitly modelled in the BN) that pollen may have been accidentally transferred from one specimen to another. The provenance of this type of data (e.g. detail of the collecting protocol) could be used to standardize data accuracy or decrease uncertainty.

#### Reasoning about vector and flower/inflorescence size and morphology

On the other hand, the relative size of the vector compared to the flower or inflorescence, and the morphology of the insect and flower/inflorescence (e.g. degree of fit, stigma accessibility), are far more difficult to elicit and represent as factors affecting the belief that a behavioral interaction or pollen transfer took place. Size is a continuous variable and the compounded nature of morphological variability is notoriously complex. Whereas knowledge of broad pollination syndromes is available [6] (e.g. flower morphology suggesting bee pollination or moth pollination, or scent suggesting fly pollination) there is no knowledge of e.g. specific morphological traits or discrete classes of vector/inflorescence size ratios that apply across all flower-visiting insect species. Specialized interactions between particular flowers and particular bees or pollen-wasps have been studied in detail to understand precisely how pollen is received and deposited [20,30].

#### Foraging behavior

The only other nodes influencing the belief in pollen transfer are the nodes representing foraging, either for pollen or oil or nectar. Again, like the degree of oligophagy, the knowledge that a given species is a pollen feeder or nectar feeder or oil collector is well established and generally available [27]. All other nodes in the BN influence the belief that one or more of these kinds of foraging or collecting behavior took place. In most records that are detailed enough to be included in an analysis, this kind of knowledge will usually determine the outcome of a BN evaluation.

### The broader relevance, to BDEI, of the elicitation and representation method

The described method of eliciting and representing biodiversity and ecological knowledge can be adapted to different perspectives on, and applications of, biodiversity science and ecology. Applying the method will be easier and the potential for success higher when the dataset units are occurrence records that include implicit or explicit knowledge about behavioral interactions between observed organisms or between organisms and the environment, e.g. in pest control (and biological control), freshwater biomonitoring, intertidal ecology, food webs (isotope analysis) or animal movement studies. Cases of implicit knowledge in databases such as host–parasite relationships and stomach–content analyses lend themselves to logical inferencing because there may be no uncertainty associated with asserting that a behavioral interaction took place between organisms (e.g. the only way that a free-living prey organism can end up in a predator’s stomach is through a predatory interaction). Similarly, enrichment of records of certain plant-insect interactions may be associated with less uncertainty than is associated with flower-visiting, particularly with e.g. obligatory leaf-miners, gallers or stem–borers. More often than not, however, behavioral interactions between organisms and the environment will need to be represented probabilistically because of inherent uncertainty and the fragmented nature of biodiversity and ecological data and knowledge. It takes time and effort to observe and record precisely how an organism is behaving, and interpret what it may be doing, and many organisms are too small or inaccessible to observe easily. Biodiversity and ecological studies are complex and data are often recorded to answer specific questions in particular ways. Nevertheless, scientists’ and natural history collections’ datasets and documents are treasure troves of incomplete data that more-or–less inadvertently and implicitly document interesting events that were not always the investigators’ intended targets.

## Conclusion and Future Work

BDEI researchers have reflected on the field’s challenges [31] and the nature of the questions that they ask of biodiversity data [32], implying that more can be achieved with natural history occurrence data than merely a display of points on a map or the use of these to predict the potential distribution of a species.

We applied knowledge engineering techniques in the context of specimen–records from natural history collections. We found that our method to elicit and represent knowledge using a semantic BN can be used to represent expert and implicit causal knowledge about ecological events so as to discover behavioral interactions in data that were collected with a different objective in mind. In future work we will focus on further developing an existing ontology, which could be combined with the semantic BN to allow both logical and probabilistic reasoning.

There is potential to use inferences about behavioral interactions between arthropods and flowers to indicate, at a higher level of biological organisation, that ecological interactions between a putative population of *species A* (arthropod) and a putative population of *species B* (plant) are to be inferred from the data. This will require aggregating the records of individual organisms into a class representing a population sample of each species. Ultimately we want to model ecological interactions (e.g. between a population of an arthropod species and a population of a plant species) relevant to flower–visiting and pollination studies using the modeling construct of an interaction network. The modelled behavioral interactions between individuals could therefore be the criteria for selecting records with which to create a network of populations linked by ecological interactions (an analogue of a community). Interaction networks are widely used in flower–visiting community ecology and studies of pollination, and standardising the concepts and automating data interpretation and construction of interaction networks could be meaningful contributions to ecological research.

## Supporting Information

S1 File Elicitation of expert knowledge. Experts were asked to read an explanation of how a Bayesian network can be used to represent knowledge, and then answer questions as to the completeness of the presented model and whether the results of running the Bayesian network using sample data were reasonable.
(DOCX)
