# Supplementary material for: Eliciting and Representing High-Level Knowledge Requirements to Discover Ecological Knowledge in Flower-Visiting Data
Source: PLoS One. 2016 Nov 16;11(11):e0166559. doi: 10.1371/journal.pone.0166559 (PMC5113002; doi:10.1371/journal.pone.0166559)
Supplement: S1 File — Experts were asked to read an explanation of how a Bayesian network can be used to represent knowledge, and then answer questions as to the completeness of the presented model and whether the results of running the Bayesian network using sample data were reasonable. (DOCX) [file pone.0166559.s001.docx]

Elicitation of expert knowledge

What is a Bayesian Network?

When constructing a Bayesian Network you are not modelling the real world (cf. ontology modelling). You are analysing the factors that affect your belief about something (usually the probability that an event took place). You are modelling the factors that influence, affect or cause an event, and you are estimating the change in your belief (probability) that the event happened according to these factors and in the light of specific evidence. A Bayesian Network merely formalizes what we automatically do every day when we ‘weigh the evidence’ for an event e.g. if your car alarm sounds you will be less concerned if there had been a thunderbolt just before you heard the alarm, or if there are several other alarms sounding, or if the car is locked safely in the garage. These factors affect your belief that the car alarm is due to a theft event. A random variable (node in the Bayesian Network) can have different states, each of which is assigned a probability or weight i.e. the chance that the event happened given that the evidence supports a particular (combination of) parent state.

The real power of a Bayesian Network is that it is based on the background / implicit knowledge that is held in the minds of experts. Unlike the domain of car alarms, the domain of flower-visiting is properly understood only by people who are experts in this field. You could argue that in a small island state with few or only very old cars, only few well-travelled people understand the domain of modern car alarms. When you build this kind of BN the exercise is therefore one of capturing the expert’s knowledge. Different experts’ views or understanding may conflict, but the idea is to build a reasonable consensus model.

A node is a random variable. An arc (arrow) is an influence. It has a direction in that A causes b, but at the same time the BN can tell us things in both directions (but it is important to distinguish cause from effect).

For example, the female sex of the bee reinforces our belief that pollen transfer (specifically that variable) took place far more than the male sex of a bee influences that belief of ours. The expert knows that it is pollen transfer, specifically, that is the ‘effect’ of the ‘cause’ (sex).

When you set the evidence nodes the Bayesian Network calculates the conditional probability of each node from all the possible combinations of states of the node’s direct parents. The network propagates this calculation throughout, resulting in the posterior probability of the ultimate node (the one whose probability we wish to estimate).

Below we see a candidate / proposed / example Bayesian Network that represents the factors affecting our belief (or ‘the probability’) that a pollination event occurred. We are in a situation in which we are examining the evidence from a natural history collection of flower-visiting insects, and we have various kinds of evidence, at the very least an insect label with a plant name written on it (But what does this mean – only an expert can tell us; or we can model the domain using a Bayesian Network). We know that a pollination event is caused by a pollen transfer event, which is caused by a FlowerProductUtilizingEvent. There are several factors that affect our belief as to whether or not a FlowerUtilizingEvent occurred (Is the plant species known to flower at the time the specimen was captured?, the degree of co-evolution displayed by the insect species’ morphology or known behaviour, the pollination syndrome (if any), and whether or not there is literature that documents flower-visiting of this plant species by this insect species). This is how an expert breaks down and weighs the evidence, but the expert is usually unaccustomed to seeing the model represented formally or explicitly.

The whole network below therefore refers to a single specimen. Imagine someone walked into your lab and presented you with a pinned bee and asked you whether it pollinated a flower. You would ask them for other evidence. The BN ‘puts the available evidence together’ in a way that simulates the mind of an expert. If we use the BN to calculate this probability (of a pollination event) for every specimen in the collection, we can more credibly claim to have constructed a ‘pollination network’ (which will consist of many BNs, one for each specimen) than someone who merely uses that label without having applied any modelling construct (which actually happens). We are not saying that we know that PollinationEvents happened. But we are saying that we have used artificial intelligence to model PollinationEvents, which may give us a result that is better than a manual effort, at least in consistency.


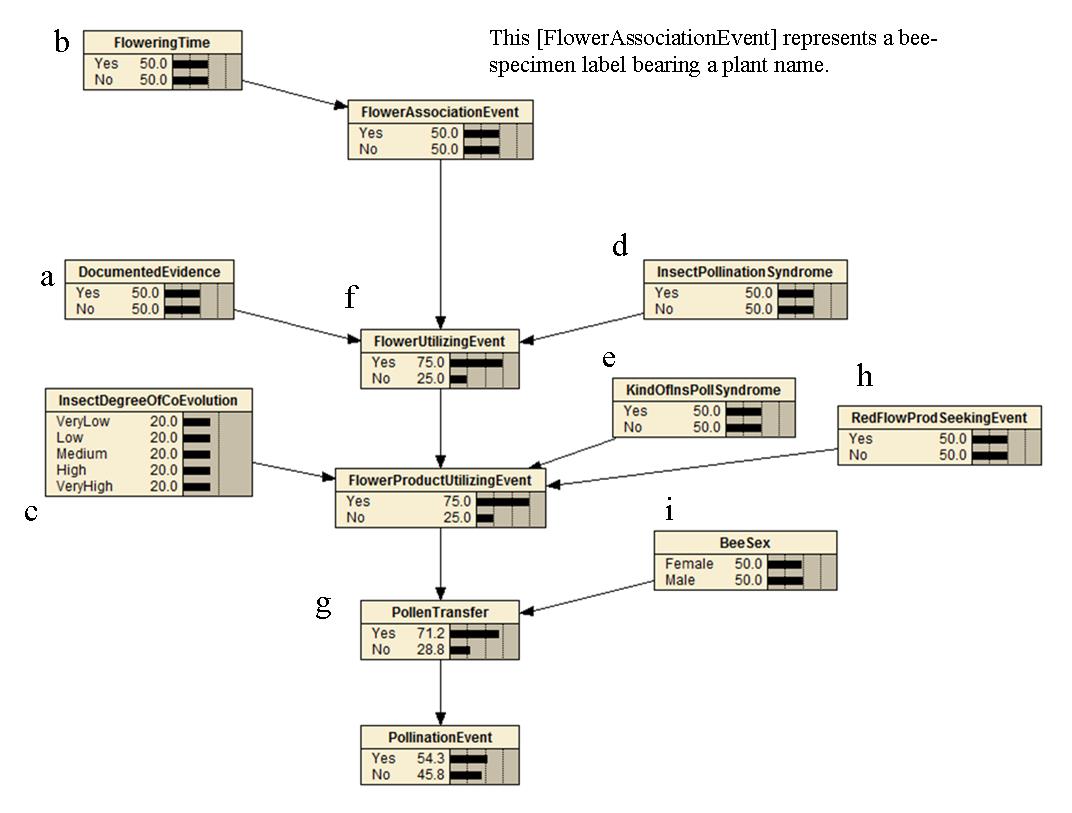


Method

I selected three specimen-records of Rediviva bees and three specimen-records of other bees. I looked at the evidence for flower-visiting in each case (i.e. on the label as well as from other sources, as depicted above).

Objective 1: Tabulate the states of the evidence nodes :

Is there documented evidence in the literature of a flower-visiting relationship between this insect species and this plant species? (NB it must not merely be a report of the insect label in question)

Is the plant species known to flower when the insect was captured?

What is the DegreeOfCoEvolution evident in the morphology or life-cycle of the insect species?

Does an insect pollination syndrome apply in the case of this plant species?

Does a SpecificPollinationSyndrome apply i.e. if it is a beetle is there evidence of a beetle pollination syndrome in the plant species?

Was a kind of a flower-visiting event observed? It is important to remember that there are two kinds of flower-visiting events: firstly there is a generalised FlowerUtilizingEvent and then there is a more specialized FlowerProductUtilizingEvent. If either of these was actually observed in the field then the evidence from all the other nodes is not as important as it would be in the absence of observational evidence of a Flower-visiting event.

Was pollen transfer observed? Perhaps we barcoded the pollen and therefore know which plant species it comes from (and if our plant specimen is of the same species we can assign a high probability that we observed pollen transfer). Here it is important to note that there may have been cross-contamination of pollen from one bee to another in the killing bottle. This can be taken into account by setting the probability to 90% rather than 100% i.e. there is a 10% chance that the evidence is spurious for one reason or another. Again, if pollen transfer was observed, the evidence from the flower-visiting node will not be as important, and the evidence from nodes leading to that node will not be as important.

Was a RedivivaFlowerProductSeekingEvent observed? i.e. the observer didn’t see the Rediviva bee collecting or ingesting oil or nectar, but noted the plant species on the label. It can therefore be inferred that the bee was seeking a floral product.

What is the sex of the bee (females require pollen protein whereas males only require the sugar in nectar and therefore may not come into contact with the pollen).

Some narrative statements to further illustrate the meaning of the random variables:

Coicidence in flowering time of the plant species and capture date of the bee specimen would reinforce my belief that this label represents a FlowerAssociationEvent, even if I only see a plant name on it and no behavioural information.

A high degree of co-evolution, evident from the bee morphology and my knowledge of what that means, reinforces my belief that a FlowerProductUtilizingEvent occurred (recall that this is when a bee uses a flower product either by ingesting it or collecting it).

The fact that the flower exhibits characteristics of a BeePollinationSyndrome, and the vector is a bee, reinforces my belief that a FlowerUtilizingEvent occurred.

Note that it is difficult to decide which variable to link the evidence to. This is the essence of modelling. Also note that the probabilities you see in the Bayesian Network above are ‘at rest’ i.e. I have not set any nodes to be evidence. Also, if a node has no parents, then the probabilities you see are prior probabilities. That is why the BeeSex has 50/50 i.e. we are saying that a given bee has a 50% chance of being female. It is only when you look at the conditional probability table of the child node (PollenTransfer) where you see the weight of being female:

Chance that pollen was transferred:

| Female bee | 80% |
| --- | --- |
| Male bee | 20% |

Objective 2: Estimate the probability of a pollination event in each case using the Bayesian Network.

| Sex | female | male | female | - | - | - |
| --- | --- | --- | --- | --- | --- | --- |
| Date | 29 Aug  1995 | 27 Aug  1987 | 13 Apr 1997 | 29 Nov 1989 | 3 April  1998 | 21 Sep  1997 |
| Insect Species | Rediviva alonsoae | Rediviva  aurata | Rediviva autumnalis | Xylocopa rufitarsis | Xylocopa senior | Xylocopa watmoughi |
| Plant Species | Alonsoa unilabiata | Diascia  elongata | Cyphia triphylla | Moraea ramosissima | Tephrosia oxygona | Sisyndite spartea |
| a) DocumentedEvidence (exclude repeats) |  | - | - | - | - | - |
| b) FloweringTime | July to Sep [Yes] | Aug to Oct  [Yes] | - | - | - | - |
| c) InsectDegreeOfCoEvolution | 5 | 5 | 5 | 3 | 3 | 3 |
| d) InsectPollinationSyndrome | Y | Y | Y | Y | Y | Y |
| e) KindOfInsPollSyndrome | Bee Pollination | Bee  Pollination | Bee  Pollination | - | - | - |
| f) FlowerVisitingEvent | RedivivaFlowerOilSeekingEvent | RedivivaFlowerProductSeekingEvent | RedivivaFlowerOilSeekingEvent | - | FlowerProductUtilizingEvent | FlowerUtilizingEvent |
| g) Pollen transfer | - | - | - | - | - | - |
| Probability of Pollination Event | 57.9 | 52 | 57.9 | 54.3 | 55 | 54.3 |
| NOTE |  | Difference is due to it being a male. Note that the ontology uses the fact that it’s a male to generalize the FlowerVisitingEvent. But the DegreeOfCoEvolution is the same as if it were a female bee. But the probability of pollen transfer is probably the same for the two kinds of flowervisitingevent. | No difference if the flowering time is not known |  | Difference between this and the one to the right is due to the kind of flowervisitingevent. |  |


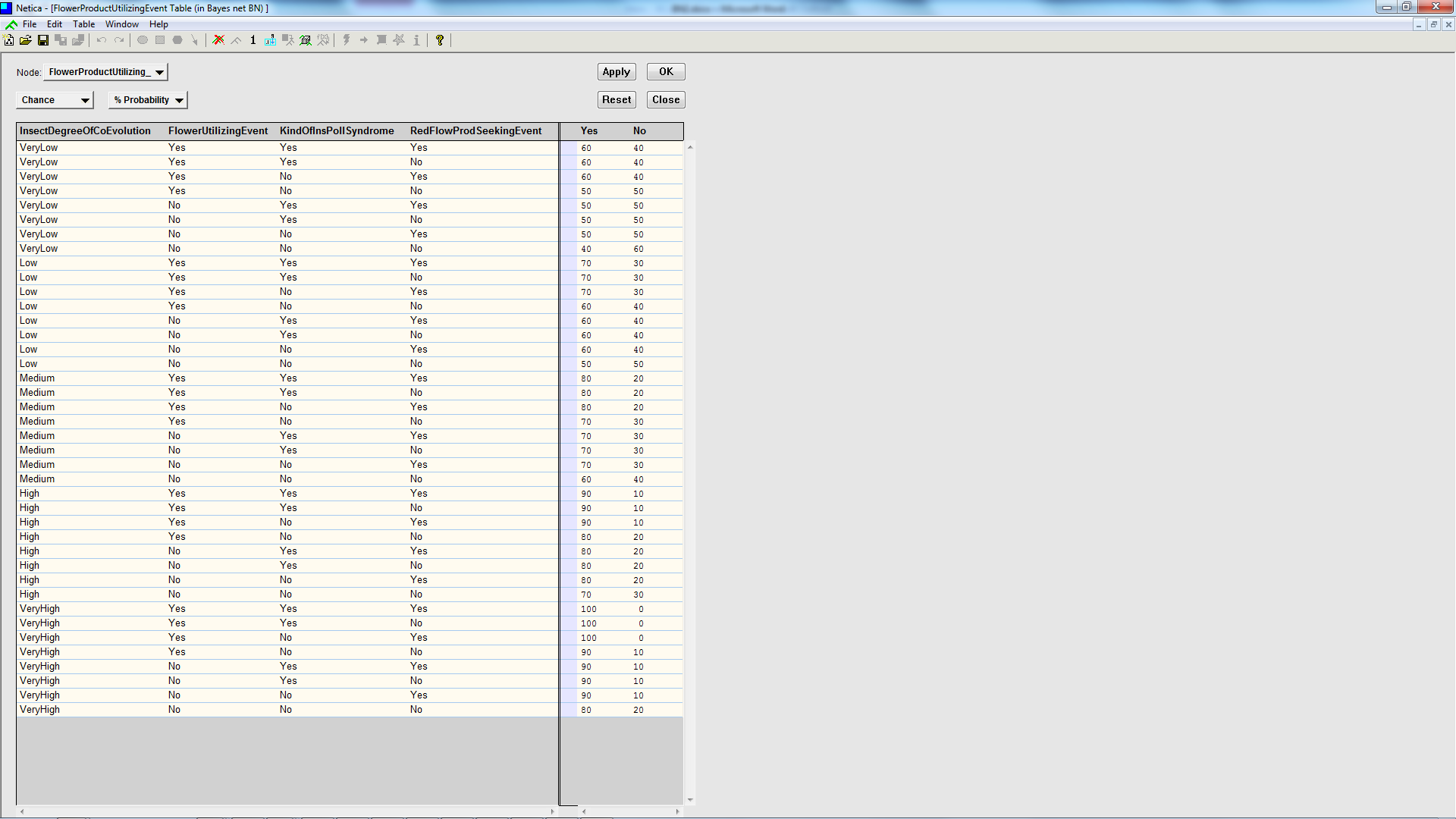


Specific questions for an expert

1) Are there any pieces of evidence (random variables / nodes) that affect your belief about the random variables depicted, which have not been considered (especially for FlowerUtilizingEvent, FlowerProductUtilizingEvent and PollenTransfer)?

2) Do you think the Conditional Probability Table for the FlowerProductUtilizingEvent, above, is reasonable? The way to read this table is as follows: We are looking at the probabilities, on the right, that a FlowerProductUtilizingEvent occurred, for each combination of parent states. Naturally the yes and no must always add up to 100.

3) Do you think the pollination event probabilities calculated for the 6 bee specimens by the Bayesian Network are reasonable?
